# Assessing the twinning model in the Rwandan Human Resources for Health Program: goal setting, satisfaction and perceived skill transfer

**DOI:** 10.1186/s12992-016-0141-4

**Published:** 2016-01-28

**Authors:** Esperance Ndenga, Glorieuse Uwizeye, Dana R. Thomson, Eric Uwitonze, Joel Mubiligi, Bethany L. Hedt-Gauthier, Michael Wilkes, Agnes Binagwaho

**Affiliations:** Human Resources for Health, Ministry of Health, P.O. Box 84, Kigali, Rwanda; College of Medicine and Health Sciences, University of Rwanda, P.O. Box 3286, Kigali, Rwanda; Department of Global Health and Social Medicine, Harvard Medical School, 641 Huntington Avenue, Boston, MA 02115 USA; Ministry of Health, P.O. Box. 84, Kigali, Rwanda; Geisel School of Medicine, 1 Rope Ferry Road, Hanover, NH 03755 USA

**Keywords:** Africa, Human resources, Health care workers, Training, Twinning, Mentorship

## Abstract

**Background:**

Because of the shortage of health professionals, particularly in specialty areas, Rwanda initiated the Human Resources for Health (HRH) Program. In this program, faculty from United States teaching institutions (USF) "twin" with Rwandan Faculty (RF) to transfer skills. This paper assesses the twinning model, exploring USF and RF goal setting, satisfaction and perceptions of the effectiveness of skill transfer within the twinning model.

**Methods:**

All USF and RF in the HRH Program from August 2012-May 2014 were invited to participate. An 85-item questionnaire for USF and 71-item questionnaire for RF were administered via Survey Monkey in April and May 2014. Associations among primary outcomes were assessed and factors related with outcomes were modeled using logistic regression.

**Results:**

Most RF and USF reported setting goals with their twin (89 % and 71 %, respectively). Half of RF (52 %) reported effective skill transfer compared to 10 % of USF. Only 38 % of RF and 28 % of USF reported being very satisfied with the twinning model. There was significant overlap in the three operational outcomes. For RF, the following factors were associated with outcomes: for effective skill transfer, being able to communicate in a common language and working at a nursing site outside of Kigali; and for satisfaction, 7+ years of professional experience and being part of a male RF-female USF twin pair. For USF, the following factors were associated with outcomes: for setting goals, prior teaching experience; and for satisfaction, experience in low resource settings for one month or less and feeling that HRH promotes a culture of respect.

**Conclusions:**

Twinning is the cornerstone of the HRH Program in Rwanda. These findings helped the HRH team identify key areas to improve the twinning experience including better recruitment and orientation of USF and RF, consideration of additional factors during the twinning process, provide language training support, facilitate joint twin activities and cross-cultural training and improve the site leadership buy-in and support of the program. These results can inform other programs using twinning to develop skills in the health workforce.

**Electronic supplementary material:**

The online version of this article (doi:10.1186/s12992-016-0141-4) contains supplementary material, which is available to authorized users.

## Background

With the established link between positive health outcomes and a sufficient number of well-qualified heath workers [1–3], developing a skilled workforce is a global priority [2]. In 2006, the World Health Organization (WHO) estimated that 4.3 million health professionals were needed globally to provide basic health services [2]. Developed countries face challenges in terms of inequitable geographical and skills distribution [4, 5]. In addition to these pressures, low and middle income countries are impacted by the migration of health workers as thousands of doctors, nurses and midwives emigrate for better work and living conditions after their countries have invested heavily in their education [6–10].

Africa, particularly sub-Saharan Africa, is highly affected by its limited health workforce. While Africa bears 24 % of the global disease burden, it employs only 3 % of healthcare workers worldwide [2]. With a staffing ratio far below the recommended 2.3 healthcare professionals per 1000 population needed to achieve the Millennium Development Goals (MDGs) [11], questions were raised as to whether countries in Africa would be able to meet the health-related MDGs [12, 13]. Indeed, while there was progress in all MDGs in sub-Saharan Africa, none of the targets were met for the region by the 2015 end-line evaluation [14].

Sub-Saharan Africa faces two critical issues: 1) the quantity/distribution of healthcare providers and 2) the skills of those providers, particularly in specialty areas. Many countries have deployed government and donor funds for long-term programs to address these challenges. In 2005, Malawi secured USD$272 million for a five-year program to train and retain health professionals [15]. In Tanzania, professional health education has evolved from one medical school in 1963 to eight universities in 2009 each containing various health-professional training programs, thus increasing the annual intake of medical students by 15 fold in the last two decades [16].

Similar to other African countries, Rwanda has a persistent shortage of qualified health professionals but the issue was heightened by the 1994 Genocide against the Tutsi where a large number of health workers were killed and others fled the country [17]. In the aftermath of the Genocide, Rwanda has invested in reconstructing the health infrastructure and workforce. As a result, the country has made dramatic progress towards achieving the health-related MDGs [18] and life expectancy has increased from 48 years in 1990 to 65 years in 2012 [19]. The only medical school in the country has increased its graduation rate from an annual average of 15 graduates in its first 25 years to five times as many in 2004–2010 [20]. Until 1998, physician specialists, in areas such as surgery, internal medicine, pediatrics, obstetrics and gynecology, were educated abroad. In 1998, the University of Rwanda created postgraduate programs to train in some of these specialty areas in country. Further, to prevent “brain drain” of health care professionals to high paying developed countries, Rwanda has implemented several strategies, including providing work contracts for health professionals in training, appointing new graduates in a timely manner and implementing performance-based financing [21].

Even with these changes, Rwanda still struggles to increase the number of healthcare professionals (doctor, nurses and midwives) to the minimum recommended by the WHO and to train health care specialists. In 2012, the Rwandan Ministry of Health, with support of the Centers for Diseases Control and Prevention and The Global Fund to Fight AIDS, Tuberculosis and Malaria, initiated the innovative 7-year Human Resources for Health (HRH) Program [21, 22]. The overall aim of HRH Program is to increase the quality of health care in Rwanda through the training of sufficient number of highly qualified health professionals capable of providing world-class care. Instead of sending Rwandan professionals to high income countries to receive specialty and subspecialty training, the HRH Program hires high caliber faculty from 23 United States teaching institutions (USI) in the fields of nursing, midwifery, medicine, dentistry, health management and public health to work in Rwanda [22]. These USI faculty (USF) are “twinned” in-country with Rwandan faculty (RF) at university and clinical teaching sites. The goal of the twinning process is to transfer skills to the RF, who in turn will continue to educate future health professionals and mentor new RF in a manner that is sustainable and cost effective.

Evidence of impact of international partnerships is limited but critical for maximizing partnership effectiveness [23]. While randomized controlled trials for such evaluations are generally not feasible, institutions engaged in partnerships are encouraged to evaluate components of the partnership and share lessons learned [24]. This paper uses RF and USF perceptions to evaluate the twinning experience, specifically in the areas of goal setting, skills transfer and participant satisfaction. These three areas were chosen because they are on the pathway to successful HRH twinning and identifying factors related to these areas can generate recommendations to improve the HRH Program and clinical training in other low-resource countries.

## Methods

### The Human Resources for Health Program in Rwanda

Since its inception in 2012, over 313 USF and 210 RF have been engaged in the HRH Program. The HRH Program targets four disciplines for RF development: medicine, nursing/midwifery, oral health and healthcare management. Each year, the University of Rwanda College of Medicine and Health Sciences (UR-CMHS) selects priorities in each discipline for specialized skills development. RF are selected at the department level at each teaching site with consideration of department goals, personal goals and skill of potential Rwandan twins.

Partner institutions in the United States screen potential USF candidates and submit these candidates for University of Rwanda consideration through the HRH team. RF and USF already based in Rwanda then advise on these applications and recommend candidates, based on their suitability for a RF twin, to subcommittees for approval. Approved USF are contracted by the respective USI for periods of time that are typically one year, except for physician sub-specialists who rotate for a minimum of 8 weeks.

Orientation is provided to USF by their home institutions prior to their departure for Rwanda. The HRH Program also organizes a pre-departure orientation phone call that focuses on programmatic and logistic matters. Once in Rwanda, HRH staff in the MOH welcome USF and introduce them to the country, and their placements, head of department and twin. Further, the HRH staff in the MOH organize a pre-orientation meeting for RF and site leadership to help them prepare to receive the USF. In August, usually within one month of the USF arrival, an annual orientation event is organized by the MOH for both USF and RF. The orientation is expected to continue at the site level by site leadership as needed. At the beginning of each year, the twin-pair is expected to set goals to be achieved during their time together; however, they are given autonomy in how they achieve these goals. HRH staff in MOH and sites leadership provide the needed support for twins to implement their goals and ensure the follow-up on progress made throughout the twinning process.

### Study design

All USF and RF engaged in the HRH Program from August 2012 to May 2014 were invited by email to respond to an online questionnaire about their perspectives and attitudes of the twinning process, program management and possible cultural and interaction challenges. The 85-item questionnaire for USF and the 71-item questionnaire for RF, administered via Survey Monkey, were identical except the USF questionnaire included additional questions about preparation and adjustment to Rwanda. The RF could opt to take the survey in either French or English, with the French version translated and back translated to English to ensure consistency in meaning between languages. Both English versions of the questionnaire were piloted among health professionals not engaged with twinning program faculty to test the flow of the question and time for completion. Data were collected in April and May 2014. Participation in the survey was optional. To increase participation, reminders were sent by the HRH staff in the MOH to all eligible participants.

### Analysis

In the study, three operational outcomes were evaluated based on HRH Program priorities: whether goals had been set, perceived effectiveness of skill transfer from USF-to-RF and satisfaction with the twinning process. A fourth outcome, time spent with twin, was originally considered as a predictor but was reconsidered as an outcome because it likely happens in tandem with goal setting skills transfer and satisfaction. It was not modeled explicitly in this paper, but was considered with other outcomes in the final models.

**Fig. 1:**
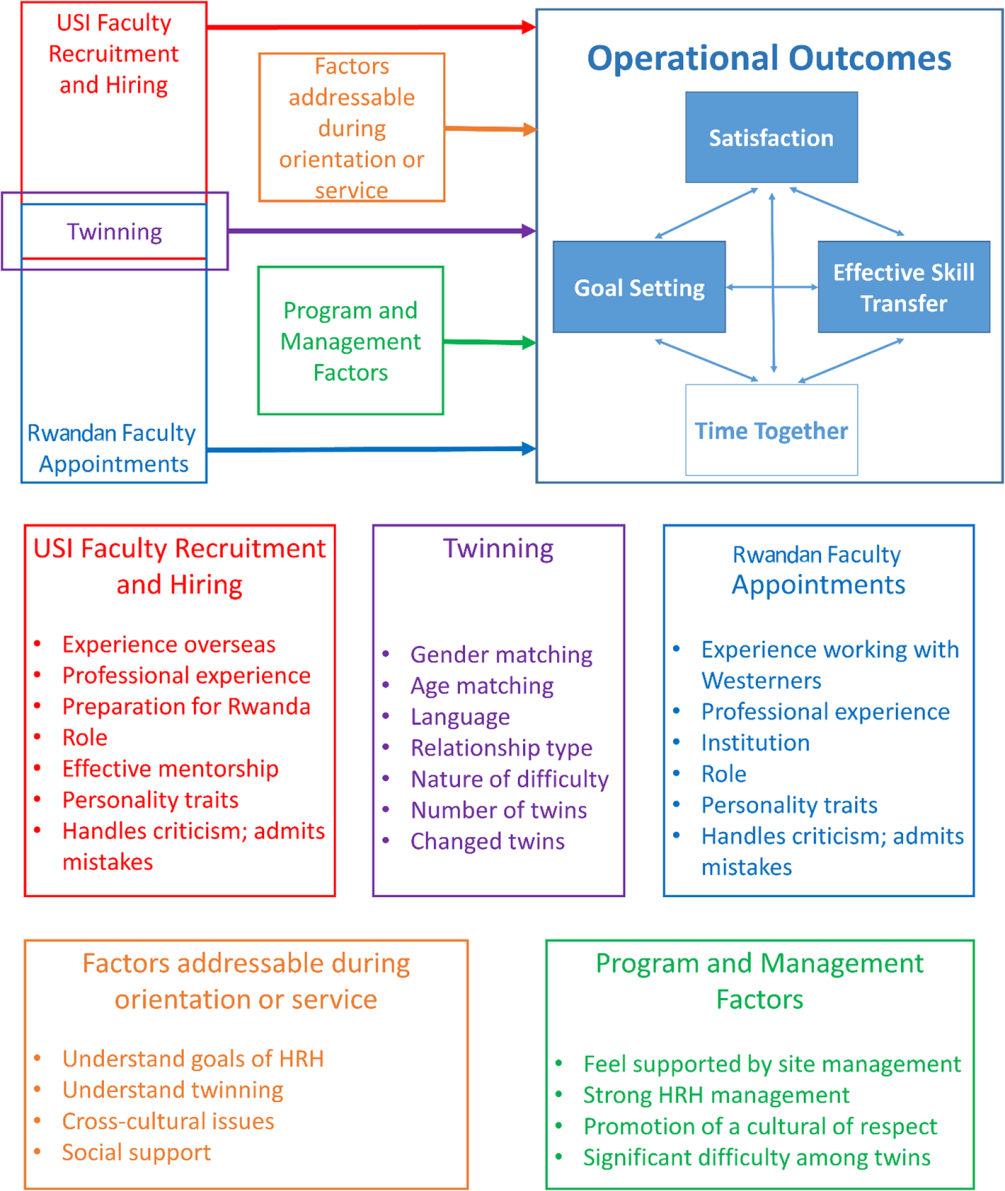
Conceptual framework of operational outcomes in the Rwandan Human Resources for Health twinning assessment

Twenty-five potential predictors were identified in a conceptual framework, grouped in the following factors (Fig. 1):

- *USF recruitment or RF appointments:* for example, time since training, previous teaching experience, experience working overseas or with Westerners before entering in the HRH Program;
- *The twinning of USF and RF*: for example, gender differences, age differences, number of twins, shared languages;
- *Factors addressable during orientation or service*: for example, understanding of HRH Program goals, understand twinning model, cultural differences; and
- *Factors related to program management*: for example, perception of support from the site leadership, feel HRH Program promotes culture of respect.

We used univariate logistic regression to test independent associations between each predictor and outcome (*p* < 0.1). Potential significant factors were considered for multivariate logistic regression models that were built using backward stepwise regression, stopping when remaining variables were associated with the outcome at the alpha = 0.1 significance level. The three primary outcomes were modeled independently and separate models were built for USF and RF, since some variables differed between the two types of faculty. Data were cleaned and analyzed in Stata version 13 (College Station, TX: StataCorp LP). We only present direction of association for statistically significant covariates; however, detailed odds ratios and *p*-values are available in Additional file 1.

### Ethics statement

Both the RF and USF surveys were reviewed and approved by HRH Program funders as part of the monitoring and evaluation plan. Since the data was collected for program purposes, the study received a waiver from the Rwandan National Ethical Committee. Participation in the survey was optional and no personal identifying information was collected.

## Results

**Fig. 2:**
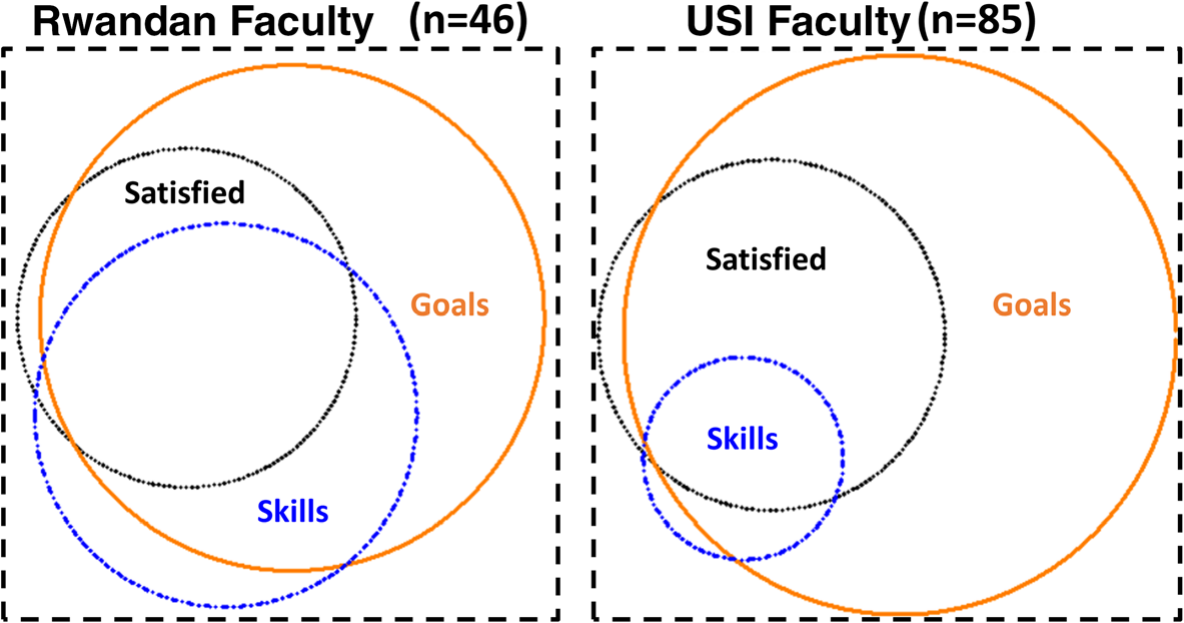
Magnitude of outcome variables and their overlap, by USI faculty and Rwandan faculty. The box represents 100 % of respondents who answered questions about all three outcomes. The size of the circle represents proportion reporting the outcome. The overlap in circles represents overlap in outcomes reported.

The response rate among RF was 49 % (*n* = 71/145) and from USF 93 % (*n* = 107/112). Among USF, 55 (49.1 %) were physicians, 36 (32.1 %) were nurses/midwives and 7 (6.3 %) were health managers (Table 1). For RF, 14 (19.7 %) were physicians, 32 (45.1 %) were nurses/midwives and 5 (7.0 %) were health managers. Seventy-three (65.2 %) of USF had 7+ years working experience post-training compared to 13 (18.3 %) of RF. Eighty percent (*n* = 57) of RF had a moderate amount of experience working with people from Western cultures before engagement in the HRH Program. Nearly half of the USF (*n* = 51, 46 %) had never worked longer than a month at a time in low-resource countries. For the three primary outcomes, there was significant overlap in prevalence of goal setting, skill transfer and feeling satisfied with the twinning experience overall for both Rwandan and USF (Fig. 2). Results for these three operational outcomes are presented below.

**Table 1:** Summary of covariates by faculty type

	Rwandan Faculty		USI Faculty	
	Number	Percent	Number	Percent
OUTCOMES				
Set joint goals with twin				
No	6	8.5	26	23.2
Yes	47	66.2	65	58.0
Missing	18	25.4	21	18.8
Very effective skill transfer (USF-to-RF)				
No	23	32.4	92	82.1
Yes	25	35.2	9	8.0
Missing	23	32.4	11	9.8
Very satisfied with twinning experience overall				
No	33	46.5	65	58.0
Yes	20	28.2	26	23.2
Missing	18	25.4	21	18.8
Number of hours per week spent working with twin				
0–9 hours	22	31.0	42	37.5
10–19 hours	12	16.9	17	15.2
20+ hours	19	26.8	32	28.6
Missing	18	25.4	21	18.8
HIRING AND APPOINTMENTS				
Discipline				
Nurse/midwife	32	45.1	36	32.1
Physician	14	19.7	55	49.1
Health manager	5	7.0	7	6.3
Lecturer, academic	20	28.2	11	9.8
Missing	0	0.0	3	2.7
Primary work site				
CHUB, CHUK, KFH, RMH, Muhima	10	14.1	18	16.1
CMHS, multiple	34	47.9	80	71.4
Nursing outside Kigali	27	38.0	14	12.5
Years since training				
7+ years	13	18.3	73	65.2
4–6 years	18	25.4	17	15.2
1–3 years	21	29.6	14	12.5
Completed prior to HRH, not yet completed	19	26.8	8	7.1
Previous teaching experience				
Moderate-little teaching	48	67.6	64	57.1
Significant teaching	23	32.4	48	42.9
Time spent in resource limited countries before HRH				
Short trips <1 month, none			51	45.5
Medium trips <6 months			17	15.2
Long trips >6 months			44	39.3
Applied talent and expertise				
Do not agree			74	66.1
Agree/strongly agree			28	25.0
Missing			10	8.9
Preparation for work in Rwanda				
Well prepared			20	17.9
Moderately prepared			50	44.6
Poorly prepared			29	25.9
Missing			13	11.6
Experience working with people from Western culture before HRH				
A lot	22	31.0		
Moderate	35	49.3		
None or minimal	14	19.7		
Twin values ALL of the following: my expertise, my opinion, our department hierarchy, my professional interests or goals				
No	45	63.4	58	51.8
Yes	2	2.8	14	12.5
Missing	24	33.8	40	35.7
Twin shows ANY of the following: withholds information, acts arrogantly, takes credit for others work, strives for power over others				
No	28	39.4	24	21.4
Yes	32	45.1	80	71.4
Missing	11	15.5	8	7.1
USI faculty is providing adequate mentorship in at least one of these roles: educator, clinician, researcher, administrator				
No	30	42.3	82	73.2
Yes	23	32.4	9	8.0
Missing	18	25.4	21	18.8
Twin handles criticism and admits mistakes quite well or extremely well				
Yes	7	9.9	10	8.9
No	5	7.0	19	17.0
Missing	59	83.1	83	74.1
TWINNING				
Number of twins ever had				
1	34	47.9	52	46.4
2	18	25.4	29	25.9
3+	12	16.9	31	27.7
Missing	7	9.9	0	0.0
Changed twins				
No change	37	52.1	54	48.2
Changed one or more times, no longer twinned	23	32.4	40	35.7
Missing	11	15.5	18	16.1
Gender differences				
Same gender	36	50.7	57	50.9
USF female, RF male	16	22.5	27	24.1
USF male, RF female	1	1.4	7	6.3
Missing	18	25.4	21	18.8
Age differences				
< 5 year difference	22	31.0	32	28.6
5–10 year difference	8	11.3	25	22.3
USF >10 years older	15	21.1	28	25.0
RF >10 years older	8	11.3	6	5.4
Missing	18	25.4	21	18.8
Ability to communicate in English, French, or Kinyarwanda				
Excellent in at least one	28	39.4	52	46.4
Moderate, fair, or poor in all	21	29.6	39	34.8
Missing	22	31.0	21	18.8
Relationship with twin				
Profession and social, other	36	50.7	39	34.8
Professional only	17	23.9	52	46.4
Missing	18	25.4	21	18.8
ORIENTATION & MANAGEMENT				
Twinning model best to achieve HRH Program goals				
No	8	11.3	39	34.8
Yes	41	57.7	28	25.0
Maybe	17	23.9	45	40.2
Missing	5	7.0	0	0.0
When understood twinning				
Once I started working / still do not understand	36	50.7	43	38.4
Before or during orientation	28	39.4	69	61.6
Missing	7	9.9	0	0.0
HRH Program goals are clear				
Yes	60	84.5	79	70.5
Missing	11	15.5	33	29.5
Nature of any difficulty with twin				
No difficulty	39	54.9	59	52.7
Task related or clinical difficulty	5	7.0	5	4.5
Time or availability difficulty	8	11.3	20	17.9
Missing	19	26.8	28	25.0
Cultural differences: % of 16 items that were ‘moderately’ or ‘extremely’ different				
Less than half moderate or extremely different	18	25.4	9	8.0
More than half moderate or extremely different	25	35.2	88	78.6
Missing	28	39.4	15	13.4
HRH Program promotes a culture of respect				
Yes	21	29.6	28	25.0
Most of the time	15	21.1	52	46.4
Rarely	7	9.9	17	15.2
Missing	28	39.4	15	13.4
Senior leadership at work site support HRH Program				
Agree or strongly agree	40	56.3	45	40.2
Disagree or strongly disagree	2	2.8	24	21.4
Neutral	1	1.4	30	26.8
Missing	28	39.4	13	11.6
Overall	71	100	112	100

### Goal setting

Among those who responded to questions about goal setting, 89 % of RF and 71 % of USF set goals (Table 1). Because none of the tested covariates were associated with goal setting among RF during bivariate analysis, a multivariate model was not built (Table 2). Among USF, a number of hiring and appointment factors (significant prior teaching experience, spent less than a month in low-resource countries at a time prior to engagement in HRH Program, feel valued by twin, twin handles criticism well), twinning factors (similar ages, excellent communication in at least one language, having a relationship that is both professional and social) and orientation or management factors (belief that HRH Program promotes a culture of respect) were associated with goal setting in bivariate analysis (*p* < 0.1) (Table 2). In multivariate analysis, significant prior teaching experience, feeling valued by one’s twin and being within 5 years of age were associated with goal setting among USF (*p* < 0.1) (Table 2).

**Table 2:** Factors associated with operational outcomes in bivariate and multivariate analysis, by faculty type

	Goals		Skills		Satisfaction	
	Rwandan Faculty	USI Faculty	Rwandan Faculty	USI Faculty	Rwandan Faculty	USI Faculty
OUTCOMES						
Very effective skill transfer (USF-to-RF)						
No					--	----
Yes					++	++++
Set joint goals with twin						
No						++
Yes						--
Very satisfied with twinning experience overall						
No		--	----	----		
Yes		++	++++	++++		
Number of hours per week spent working with twin						
0–9 hours		----			----	----
10–19 hours					++	
20+ hours		++++			++++	++++
HIRING AND APPOINTMENTS						
Primary work site						
CMHS, multiple			----			
CHUB, CHUK, KFH, RMH, Muhima						
Nursing outside Kigali			++++			
Years since training						
7+ years			++		++++	
4–6 years					----	
1–3 years			--		--	
Completed prior to HRH, not yet completed					--	
Previous teaching experience						
Moderate-little teaching		----				
Significant teaching		++++				
Time spent in resource limited countries before HRH Program						
Short trips <1 month, none		++				++++
Medium trips <6 months		--				----
Long trips >6 months						----
Applied talent and expertise						
Agree, strongly agree						++
Do not agree						--
Experience working with people from Western culture before HRH Program						
A lot					++	
Moderate					--	
None or minimal						
Twin values ALL of the following: my expertise, my opinion, our department hierarchy, my professional interests or goals						
No		++++				
Yes		----				
USI faculty is providing adequate mentorship in at least one of these roles: educator, clinician, researcher, administrator						
No						--
Yes						++
TWINNING						
Gender differences						
Same gender					----	
USF female-RF male					++++	
USF male-RF female						
Age differences						
< 5 year difference		++++				
5–10 year difference		----				
USF >10 years older						
RF >10 years older						
Ability to communicate in English, French, or Kinyarwanda						
Excellent in at least one		++	++++			++
Moderate, fair, or poor in all		--	----			--
Relationship with twin						
Profession and social, other		++		++		++
Professional only		--		--		--
Twin handles criticism and admits mistakes quite well or extremely well						
No		++				++
Yes		--				--
ORIENTATION & MANAGEMENT						
Twinning model best to achieve HRH Program goals						
No						--
Yes						++
HRH Program promotes a culture of respect						
Yes		++		++		++++
Most of the time		--		--		----
Rarely		--				
Senior leadership at work site support HRH Program						
Agree or strongly agree						++++
Disagree or strongly disagree						----
Neutral						--

Code: -- indicates significantly lower on univariable, ---- indicates significantly lower in multivariable; ++ indicates significantly higher on univariable ++++ indicates significantly higher in multivariable

### Skills transfer

Among those who responded to questions about skill transfer, half (52 %) of RF reported “very effective” skill transfer from their USF twin, whereas less than 10 % of USF felt they transferred skills very effectively to their RF twin (Table 1). In bivariate analysis of RF, three factors were significantly associated with skill transfer (at *p* < 0.1): participating in a nursing program outside of Kigali compared to the main university campus or working at multiple hospitals, being seven or more years post training and having excellent communication with twin in one or more languages (Table 2). Sharing a common language and being in a nursing program outside of Kigali remained significant in multivariate regression (*p* < 0.1) (Table 2). Among USF, sharing a professional and social relationship with their twin and believing that HRH Program promotes a culture of respect were associated with perceived skill transfer to a Rwandan twin in bivariate analysis (*p* < 0.1), though neither factor remained significant in multivariate analysis when satisfaction was in the model (*p* < 0.1) (Table 2).

### Satisfaction

Among those who answered questions about satisfaction, 38 % of RF and 28 % of USF reported they were “very satisfied” with the twinning experience overall (Table 1). In bivariate analysis, factors associated with RF satisfaction were: being seven or more years post training, having a lot of experience with people from Western culture before engagement in the HRH Program and being a male RF twinned with a female USF versus same gender pairing (*p* < 0.1) (Table 2). Being seven years post training and male RF-female USF pairs remained significant in multivariate analysis (*p* < 0.1). Among USF in bivariate analysis, satisfaction was predicted by a number of hiring or appointment factors (spent less than a month in low-resource countries at a time prior to engage in HRH Program, feel he/she is applying own talent and expertise, feel he/she is adequately mentoring others in educator, clinical, research, or administrator roles, and twin handles criticism well), twinning factors (have excellent communication in at least one language, share a professional and social relationship with twin) and orientation or management factors (belief that twinning model is the best way to achieve HRH Program goals, feel that HRH Program promotes a culture of respect, and the senior leadership at sites support the HRH Program) (*p* < 0.1) (Table 2). In multivariate analysis, having prior experiences of less than a month in low-resource countries and feeling the HRH Program promotes a culture of respect and work site senior leadership support the HRH Program remained significant (*p* < 0.1).

## Discussion

In this survey, the link between satisfaction, spending time with HRH twin, goal setting and perceived skill transfer was very strong. The highest performing outcome was goal setting, with 89 % of RF and 71 % of USF reporting that they had set joint goals with their twin. While the proportion of faculty who were “very satisfied” was only 29 % among USF and 38 % among RF, most faculty (93 % RF, 59 % USF) expressed satisfaction to some extent. Central to the overall mission of the HRH Program, the lowest performing outcome was effective skill transfer; interestingly, this result was disparate between the RF and USF with 50 % and 10 % reporting effective skill transfer, respectively. It should be noted that all items were self-reported perceptions and all, particularly the effective skill transfer, could be based on cultural or historical expectations such that what RF or USF faculty believe to be effective skills transfer may vary. The findings of important factors that may improve RF and USF twinning experiences are described in more detail below.

### Recruitment and onboarding of US institution faculty

We were surprised to find that USF with the most overseas experience were the least satisfied with the HRH Program. We assumed extensive prior work overseas would help the faculty set expectations and prepare for their engagement in HRH Program experience. However, the HRH Program uses a unique model with the USF working on a country-developed agenda under the leadership of Rwandans. We suspect this is different from other programs where the USF may have mostly held leadership positions and developed their own workplan. Indeed, previous studies have shown that satisfaction among working professionals in Western settings is closely linked with decision-making ability on the job [25]. As such, the expectations drawn from the USF’s prior extensive overseas experience may not have been met during their work with the HRH Program.

We are certainly not suggesting that in future years the HRH Program should not recruit USF with previous international experience. However, we do think sufficient pre-Rwanda training, focusing on linking faculty’s experiences to what can be expected from their time in Rwanda will be key for managing expectations and increasing overall satisfaction [26]. Further, the orientation should include clear information on the HRH Program and more details on work expectations, the twinning model, the health and education systems in Rwanda as well as cross-cultural matters to better prepare all USF for their engagement in HRH work.

### Recruitment and onboarding of Rwandan faculty

Compared to most professional development programs that focus on new faculty [27], the HRH Program targets RF of various levels. In this survey, RF with more than seven years of experience reported the highest level of skill transfer and were more likely to be satisfied by the twinning experience. In other studies in the United States, senior faculty showed interest in professional development programs such as mentorship [28, 29]; while the Rwandan context has marked differences, we believe that similarly, more experienced faculty will value the opportunity to learn from USF and will be better able to articulate concrete learning goals for the twinning process.

We hypothesize the finding that less experienced Rwandan colleagues were less satisfied and perceived less effective skill transfer may also reflect two challenges that were observed in studies from the United States: 1) low motivation because they are unclear how the gain of skills through the HRH Program will tangibly lead to personal and professional promotion [29] and 2) lack of time to participate. In the future, if these faculty are selected as targets for the HRH Program, then we recommend allocating dedicated time to participate and to award their participation during faculty performance appraisal and with credit for continuing medical education [27].

### Twinning matters

The twinning model creates a framework in which skills are transferred primarily to the RF, and thus how twins are paired is important to the success of the program. The best way to match twins is not yet established. Some programs assign pairs [27] and other programs allowing pairs to self-identify [28, 30]. For the HRH Program, each department has generally assigned pairs, weighing the training needs of the RF with the skills of the USF. However, we note from these survey results that the twinning process should also consider the age, gender and language competencies of the twins. We found that twins of approximately the same age more likely to set goals together, which is consistent with other programs that found large age differences may promote a hierarchy that prevents effective learning in junior faculty [29]. Unexpectedly, male RF reported more satisfaction when matched with female USF. This is contrary to other studies that recommend same-gender pairs [28] and should be explored more in future surveys. Inability to communicate in the same language was linked to lower likelihood of setting goals and lower satisfaction in USF and lower likelihood to report effective skill transfer in RF. Since many USF are not fluent in French and very few speak Kinyarwanda, ideally the initiation of RF should make them fluent in English, the official language of Rwanda since 2011. We will also encourage USF to take French lessons prior the joining the twinning program, and as time and resources allow, provide some French training within the HRH Program.

### The role of cross-cultural training and exchanges

In addition to professional relationships with the twin, it turns out that social relationships are also important. Social connections would allow USF to feel more integrated, to gain a deeper understanding of their twin and to understand some of the cultural content surrounding medical practice [31–33]. We also believe that other factors identified in this study could be linked to cross-cultural misunderstandings. RF with more experience working with someone from Western cultures were more likely to be satisfied with the HRH Program. Further, USF who felt that their expertise or opinions were not valued were less likely to set goals and USF who felt their twin did not handle criticism well were less likely to set goals and be satisfied with the HRH Program. We hypothesize that perception of how others value expertise or handle criticism may be influenced by cultural lenses, and that the perception of these characteristics could be improved with cross-cultural sensitization on Rwandan-Western cultural norms. In future years, the HRH Program plans to integrate cross-cultural training into RF and USF orientations, and to nurture social relationships and cross-cultural exchanges through sponsored non-work activities.

### Modeling effective programs

Nursing sites outside of Kigali were linked to more effective skill transfer, and the reasons are not entirely clear. One possible explanation is that the nursing programs outside Kigali are located in semi-urban areas where interactions among nursing faculty more easily blend social and professional life compared to faculty located in Kigali or assigned to multiple teaching sites. Further, RF at these nursing sites are often enrolled in formal training and degree upgrading programs, so they may benefit from their twin both directly through HRH Program activities and through informal support for their work in these training programs. We recommend further investigation to understand why skill transfer at nursing sites outside of Kigali was perceived to be so effective, which might improve skill transfer at teaching hospitals and the university located in Kigali.

### HRH Program buy-in at the institutional level

To make twinning more successful and gratifying for USF and RF, HRH Program leadership both at central and teaching sites should be actively involved with creating collaborative work environments. USF who felt supported by site leadership were more satisfied with the twinning process, and we believe that this is linked to receiving support needed to achieve twinning goals. The fact that some site leaders were not heavily engaged in the initial HRH years is a function of the limited human resources at the health institution leadership level, such that they were unable to provide necessary support or resources due to competing institutional demands. To address this, the HRH Program has started a series of meetings with UR-CMHS and department leadership to discuss challenges and provide solutions to overcome potential barriers for effective twinning and skills transfer at site.

#### Limitations

Several limitations should be considered in the interpretation of these results. The present study targeted all faculty who participated in the program in the period between August 2012 and May 2013; however, it is possible that we were unable to invite some faculty as a result of our inability to make contact with them such as having an incorrect email address. Further, RF participation in the survey was lower compared with USF participation. This may have been due the fact that both clinical and classroom teaching work kept them busy such that finding time to participate in the study was difficult. In addition the use of an online survey may have reduced the number of RF responding due to limited previous exposure to online surveys and poor internet access. The questionnaire was long, which may have increased missing rates for later questions. The survey may have had recall biases, particularly for faculty who had not been in the HRH Program for over a year. Finally, although respondents were assured that no directly identifiable information would be collected, respondents who had unique demographic or job profiles in the program may have been less forthcoming with criticism if they felt responses could not be anonymized. However, despite these limitations, we believe these results are informative, both because of the uniqueness of the twinning model in the HRH Program and because this is the first formal assessment of the satisfaction and perceived effectiveness of this program. We intend to follow-up this assessment with RF and USF interviews to corroborate these findings and to better understand some of the associations observed.

## Conclusions

Overall, the HRH Program has been useful for improving the skills of the health workforce in Rwanda. However, central to the HRH Program is the twinning model, specifically goal setting and skills transfer between twins, and satisfaction with the twinning model is important for the HRH Program success. The findings of this study helped the HRH team identify key areas to improve the twinning experience. For example, following information gained from this survey, the program has improved pre-orientation of USF and RF, it is increasing its staff support to assist cross-cultural training and social activities, and studying best practices of the nursing sites. Other factors such as age and gender in the matching process need more investigation, but the HRH team is willing to advise on considering these factors in the matching of RF-USF twins if supported by the evidence.

To ensure continuous improvement of twinning model, mechanisms to collect feedback on the twinning process will be reinforced to expand best practice and manage reported challenges. This will both optimize the HRH Program in Rwanda and provide information that can contribute to the development of health workforces in other low-income countries through implementation of effective models.

## Additional file

Additional file 1:
**Univariate and multivariate associations between all covariates and outcomes, by faculty type.** (DOCX 77 kb)
