# Supplementary material for: Assessing the twinning model in the Rwandan Human Resources for Health Program: goal setting, satisfaction and perceived skill transfer
Source: Global Health. 2016 Jan 28;12:4. doi: 10.1186/s12992-016-0141-4 (PMC4730618; doi:10.1186/s12992-016-0141-4)
Supplement: Additional file 1: — Univariate and multivariate associations between all covariates and outcomes, by faculty type. (DOCX 77 kb) [file 12992_2016_141_MOESM1_ESM.docx]

**Additional File 1.** Univariate and Multivariate associations between all covariates and outcomes, by faculty type

|  | RF no goals † | USF no goals † | | RF Skill transfer | | USF Skill transfer | | RF satisfaction | | USF satisfaction | |
| --- | --- | --- | --- | --- | --- | --- | --- | --- | --- | --- | --- |
|  | Uni. | Uni. | Multi. | Uni. | Multi. | Uni. | Multi. | Uni. | Multi. | Uni. | Multi. |
| **Overall** |  |  |  |  |  |  |  |  |  |  |  |
| **Very effective skill transfer (USF-to-RF)** |  |  |  |  |  |  |  |  |  |  |  |
| No | 1.00 | 1.00 |  |  |  |  |  | 1.00 |  | 1.00 | 1.00 |
| Yes | 1.00 | Omit |  |  |  |  |  | 4.68* |  | 9.83** | 27.74* |
| Missing | omit | 1.10 |  |  |  |  |  | 1.44 |  | 1.64 | 1.14 |
| **Set joint goals with twin** |  |  |  |  |  |  |  |  |  |  |  |
| Yes |  |  |  | 1.00 |  | 1.00 |  | 1.00 |  | 1.00 |  |
| No |  |  |  | 1.00 |  | omit |  | omit |  | 0.06** |  |
| Missing |  |  |  | Omit |  | 0.44 |  | omit |  | omit |  |
| **Very satisfied with twinning experience overall** |  |  |  |  |  |  |  |  |  |  |  |
| No | 1.00 | 1.00 |  | 1.00 | 1.00 | 1.00 | 1.00 |  |  |  |  |
| Yes | omit | 0.06** |  | 4.68* | 7.31* | 9.83** | 9.83** |  |  |  |  |
| Missing | omit | Omit |  | Omit | omit | 1.97 | 1.97 |  |  |  |  |
| **Number of hours per week spent working with twin** |  |  |  |  |  |  |  |  |  |  |  |
| 0-9 hours | 1.00 | 1.00 | 1.00 | 1.00 |  | 1.00 |  | 1.00 | 1.00 | 1.00 | 1.00 |
| 10-19 hours | 2.44 | 1.03 | 2.10 | 1.65 |  | 4.62 |  | 4.50* | 3.05 | 1.29 | 0.97 |
| 20+ hours | 4.00 | 0.10** | 0.13* | 1.77 |  | 1.98 |  | 5.00* | 7.90* | 6.80** | 9.40** |
| Missing | omit | Omit | omit | Omit |  | 1.23 |  | omit | omit | omit | omit |
| **Discipline** |  |  |  |  |  |  |  |  |  |  |  |
| Nurse, midwife | 1.00 | 1.00 |  | 1.00 |  | 1.00 |  | 1.00 |  | 1.00 |  |
| Physician | 1.36 | 2.25 |  | 0.68 |  | 1.67 |  | 0.86 |  | 0.54 |  |
| Health manager | 0.41 | 3.50 |  | 0.85 |  | 3.00 |  | 1.50 |  | omit |  |
| Lecturer, academic | 1.64 | 3.50 |  | 1.02 |  | 1.67 |  | 0.67 |  | 0.24 |  |
| Missing | omit | 2.33 |  | Omit |  | omit |  | omit |  | omit |  |
| **Primary work site** |  |  |  |  |  |  |  |  |  |  |  |
| CMHS, multiple | 1.00 | 1.00 |  | 1.00 | 1.00 | 1.00 |  | 1.00 |  | 1.00 |  |
| CHUB, CHUK, KFH, RMH, Muhima | 1.64 | 0.67 |  | 6.00 | 6.42 | 1.75 |  | 1.11 |  | 2.40 |  |
| Nursing outside Kigali | 0.81 | 0.72 |  | 13.00* | 41.03* | 1.33 |  | 0.90 |  | 4.50 |  |
| **Years since training** |  |  |  |  |  |  |  |  |  |  |  |
| 7+ years | 1.00 | 1.00 |  | 1.00 |  | 1.00 |  | 1.00 | 1.00 | 1.00 |  |
| 4-6 years | 1.00 | 1.10 |  | 0.27 |  | 3.52 |  | 0.03** | 0.03* | 0.27 |  |
| 1-3 years | 2.50 | 0.48 |  | 0.15* |  | 2.77 |  | 0.16* | 0.07* | 0.38 |  |
| Completed prior to HRH, not yet completed | omit | 1.61 |  | 1.20 |  | omit |  | 0.17* | 0.29 | 1.27 |  |
| **Previous teaching experience** |  |  |  |  |  |  |  |  |  |  |  |
| Moderate-little teaching | 1.00 | 1.00 | 1.00 | 1.00 |  | 1.00 |  | 1.00 |  | 1.00 |  |
| Significant teaching | 0.42 | 0.31* | 0.14* | 0.41 |  | 0.33 |  | 0.86 |  | 1.60 |  |
| **Time spent in resource limited countries before HRH Program** |  |  |  |  |  |  |  |  |  |  |  |
| Short trips <1 month, none |  | 1.00 |  |  |  | 1.00 |  |  |  | 1.00 | 1.00 |
| Medium trips <6 months |  | 3.14* |  |  |  | 0.70 |  |  |  | 0.12* | 0.08 |
| Long trips >6 months |  | 1.61 |  |  |  | 1.00 |  |  |  | 0.42* | 0.25* |
| **Applied talent and expertise** |  |  |  |  |  |  |  |  |  |  |  |
| Agree, strongly agree |  | 1.00 |  |  |  | 1.00 |  |  |  | 1.00 |  |
| Do not agree |  | 2.04 |  |  |  | omit |  |  |  | 0.22* |  |
| Missing |  | 1.53 |  |  |  | omit |  |  |  | 0.98 |  |
| **Preparation for work in Rwanda** |  |  |  |  |  |  |  |  |  |  |  |
| Well prepared |  | 1.00 |  |  |  | 1.00 |  |  |  | 1.00 |  |
| Moderately prepared |  | 0.43 |  |  |  | 0.34 |  |  |  | 1.14 |  |
| Poorly prepared |  | 1.15 |  |  |  | 0.62 |  |  |  | 0.35 |  |
| Missing |  | 0.48 |  |  |  | omit |  |  |  | 1.10 |  |
| **Experience working with people from Western culture before HRH Program** |  |  |  |  |  |  |  |  |  |  |  |
| A lot | 2.91 |  |  | 0.49 |  |  |  | 0.56 |  |  |  |
| Moderate | 2.40 |  |  | 1.07 |  |  |  | 0.31* |  |  |  |
| None or minimal | 1.00 |  |  | 1.00 |  |  |  | 1.00 |  |  |  |
| **Twin values ALL of the following: my expertise, my opinion, our department hierarchy, my professional interests or goals** |  |  |  |  |  |  |  |  |  |  |  |
| Yes | 1.00 | 1.00 | 1.00 | 1.00 |  | 1.00 |  | 1.00 |  | 1.00 |  |
| No | omit | 9.58** | 6.48* | omit |  | omit |  | 1.50 |  | omit |  |
| Missing | 0.62 | 1.02 | 0.65 | 2.10 |  | 0.40 |  | 0.30 |  | 0.63 |  |
| **Twin shows ANY of the following: withholds information, acts arrogantly, takes credit for others work** |  |  |  |  |  |  |  |  |  |  |  |
| Yes | 1.00 | 1.00 |  | 1.00 |  | 1.00 |  | 1.00 |  | 1.00 |  |
| No | 0.60 | 0.17 |  | 1.17 |  | 2.15 |  | 1.60 |  | 1.29 |  |
| Missing | omit | 0.50 |  | 2.92 |  | omit |  | 1.33 |  | omit |  |
| **USI faculty is providing adequate mentorship in at least one of these roles: educator, clinician, researcher, administrator** |  |  |  |  |  |  |  |  |  |  |  |
| No | 1.00 | 1.00 |  | 1.00 |  | 1.00 |  | 1.00 |  | 1.00 |  |
| Yes | 1.62 | 0.28 |  | 2.10 |  | 1.69 |  | 2.14 |  | 3.63* |  |
| Missing | omit | omit |  | omit |  | 0.68 |  | omit |  | omit |  |
| **Number of twins ever had** |  |  |  |  |  |  |  |  |  |  |  |
| 1 | 1.00 | 1.00 |  | 1.00 |  | 1.00 |  | 1.00 |  | 1.00 |  |
| 2 | 0.42 | 1.48 |  | 0.63 |  | omit |  | 0.65 |  | 0.92 |  |
| 3+ | omit | 0.81 |  | 0.59 |  | 0.78 |  | 1.64 |  | 0.84 |  |
| **Changed twins** |  |  |  |  |  |  |  |  |  |  |  |
| No change | 1.00 | 1.00 |  | 1.00 |  | 1.00 |  | 1.00 |  | 1.00 |  |
| Changed one or more times, not longer twinned | omit | 1.87 |  | 0.68 |  | 0.40 |  | 0.75 |  | 0.78 |  |
| Missing | omit | omit |  | omit |  | 0.47 |  | omit |  | omit |  |
| **Gender differences** |  |  |  |  |  |  |  |  |  |  |  |
| Same gender | 1.00 | 1.00 |  | 1.00 |  | 1.00 |  | 1.00 | 1.00 | 1.00 |  |
| USF female-RF male | 0.41 | 1.08 |  | 0.58 |  | 0.27 |  | 3.34* | 3.98* | 0.49 |  |
| USF male-RF female | omit | 1.02 |  | 1.00 |  | 1.00 |  | omit | omit | 1.63 |  |
| Missing | omit | omit |  | 1.00 |  | 0.45 |  | omit | omit | omit |  |
| **Age differences** |  |  |  |  |  |  |  |  |  |  |  |
| <5 year difference | 1.00 | 1.00 | 1.00 | 1.00 |  | 1.00 |  | 1.00 |  | 1.00 |  |
| 5-10 year difference | 0.33 | 3.87* | 12.08* | 1.00 |  | omit |  | 2.41 |  | 0.49 |  |
| USF >10 years older | 0.31 | 0.78 | 1.79 | 0.88 |  | 0.57 |  | 0.72 |  | 1.92 |  |
| RF >10 years older | 0.14 | 0.71 | 1.33 | 1.33 |  | omit |  | 0.21 |  | 0.51 |  |
| Missing | omit | omit |  | omit |  | 0.31 |  | omit |  | omit |  |
| **Ability to communicate in English, French, or Kinyarwanda** |  |  |  |  |  |  |  |  |  |  |  |
| Excellent in at least one | 1.00 | 1.00 |  | 1.00 | 1.00 | 1.00 |  | 1.00 |  | 1.00 |  |
| Moderate, fair, or poor in all | 1.14 | 2.33* |  | 0.33* | 0.19* | 0.36 |  | 0.58 |  | 0.38* |  |
| Missing | 0.36 | omit |  | 0.56 | omit | 0.44 |  | omit |  | omit |  |
| **Relationship with twin** |  |  |  |  |  |  |  |  |  |  |  |
| Profession and social, other | 1.00 | 1.00 |  | 1.00 |  | 1.00 |  | 1.00 |  | 1.00 |  |
| Professional only | 0.94 | 2.63* |  | 0.36 |  | 0.20* |  | 0.86 |  | 0.16*** |  |
| Missing | omit | omit |  | omit |  | 0.32 |  | omit |  | omit |  |
| **Twin handles criticism and admits mistakes quite well or extremely well** |  |  |  |  |  |  |  |  |  |  |  |
| Yes | 1.00 | 1.00 |  | 1.00 |  | 1.00 |  | 1.00 |  | 1.00 |  |
| No | omit | 3.19* |  | 0.75 |  | omit |  | omit |  | 0.04** |  |
| Missing | 1.20 | omit |  | 0.79 |  | 1.12 |  | 0.48 |  | 0.29* |  |
| **Twinning model best to achieve HRH Program goals** |  |  |  |  |  |  |  |  |  |  |  |
| No | 1.00 | 1.00 |  | 1.00 |  | 1.00 |  | 1.00 |  | 1.00 |  |
| Yes | 2.14 | omit |  | 2.83 |  | 4.25 |  | 4.79 |  | 23.7*** |  |
| Maybe | 0.48 | 1.38 |  | 1.71 |  | 0.89 |  | 6.00 |  | 0.54 |  |
| **When understood twinning** |  |  |  |  |  |  |  |  |  |  |  |
| Once I started working, still do not understand | 1.00 | 1.00 |  | 1.00 |  | 1.00 |  | 1.00 |  | 1.00 |  |
| Before or during orientation | 4.40 | 0.48 |  | 1.01 |  | 5.63 |  | 1.54 |  | 1.93 |  |
| **HRH Program goals are clear** |  |  |  |  |  |  |  |  |  |  |  |
| Yes | 1.00 | 1.00 |  | 1.00 |  | 1.00 |  | 1.00 |  | 1.00 |  |
| Missing | 0.47 | 1.38 |  | 0.28 |  | 1.20 |  | omit |  | 0.83 |  |
| **Nature of any difficulty with twin** |  |  |  |  |  |  |  |  |  |  |  |
| No difficulty | 1.00 | 1.00 |  | 1.00 |  | 1.00 |  | 1.00 |  | 1.00 |  |
| Task related or clinical difficulty | omit | 1.96 |  | 2.12 |  | omit |  | 0.36 |  | 0.57 |  |
| Time or availability difficulty | 1.03 | 0.98 |  | 1.06 |  | 0.35 |  | 0.86 |  | 0.76 |  |
| Missing | omit | 3.91* |  | omit |  | 0.32 |  | omit |  | 0.91 |  |
| **Cultural differences: % of 16 items that were 'moderately' or 'extremely' differ** |  |  |  |  |  |  |  |  |  |  |  |
| Less than half moderate or extremely different | 1.00 | 1.00 |  | 1.00 |  | 1.00 |  | 1.00 |  | 1.00 |  |
| More than half moderate or extremely different | 0.31 | 0.36 |  | 1.35 |  | 0.30 |  | 1.02 |  | 0.36 |  |
| Missing | 0.69 | 0.43 |  | 5.00 |  | omit |  | 0.48 |  | 0.43 |  |
| **HRH promotes a culture of respect** |  |  |  |  |  |  |  |  |  |  |  |
| Yes | 1.00 | 1.00 |  | 1.00 |  | 1.00 |  | 1.00 |  | 1.00 | 1.00 |
| Most of the time | 0.23 | 3.45* |  | 0.41 |  | 0.21* |  | 0.68 |  | 0.24* | 0.19* |
| Rarely | 0.55 | 5.00* |  | 0.25 |  | omit |  | 0.92 |  | 0.44 | 1.49 |
| Missing | omit | 1.67 |  | 2.46 |  | omit |  | 0.41 |  | 0.47 | omit |
| **Senior leadership at worksite support HRH Program** |  |  |  |  |  |  |  |  |  |  |  |
| Agree or strongly agree | 1.00 | 1.00 |  | 1.00 |  | 1.00 |  | 1.00 |  | 1.00 | 1.00 |
| Disagree or strongly disagree | omit | 2.11 |  | omit |  | 0.28 |  | 1.37 |  | 0.15* | 0.15* |
| Neutral | omit | 1.92 |  | omit |  | 0.45 |  | omit |  | 0.31* | 0.26 |
| Missing | 1.67 | 1.04 |  | 4.89 |  | omit |  | 0.46 |  | 0.66 | omit |

† Goal setting reference is "yes set goals" to have a non-zero reference category; "no goal set" has zero observations in some models. Therefore direction of association is opposite of other models in bivariate and multivariate models
